# Improved diagnosis and prognostication of patients with pleural malignant mesothelioma using biomarkers in pleural effusions and peripheral blood samples – a short report

**DOI:** 10.1007/s13402-017-0327-7

**Published:** 2017-06-02

**Authors:** Nick Beije, Jaco Kraan, Michael A. den Bakker, Alexander P.W.M. Maat, Cor van der Leest, Robin Cornelissen, Ngoc M. Van, John W.M. Martens, Joachim G.J.V. Aerts, Stefan Sleijfer

**Affiliations:** 1 Department of Medical Oncology and Cancer Genomics Netherlands, Erasmus MC Cancer Institute, Erasmus University Medical Center, Wytemaweg 80, 3015 CN Rotterdam, The Netherlands; 2 Department of Pathology, Erasmus MC Cancer Institute, Erasmus University Medical Center, Rotterdam, The Netherlands; 3 Department of Pathology, Maasstad Hospital, Rotterdam, The Netherlands; 4 Department of Cardiothoracic Surgery, Erasmus MC Cancer Institute, Erasmus University Medical Center, Rotterdam, The Netherlands; 5 Department of Pulmonary Medicine, Amphia Hospital, Breda, The Netherlands; 6 Department of Pulmonary Medicine, Erasmus MC Cancer Institute, Erasmus University Medical Center, Rotterdam, The Netherlands

**Keywords:** Malignant pleural mesothelioma, Pleural effusion, Circulating tumor cells, Circulating endothelial cells, Tumor endothelial marker

## Abstract

**Purpose:**

There is a lack of robust and clinically utilizable markers for the diagnosis and prognostication of malignant pleural mesothelioma (MPM). This research was aimed at optimizing and exploring novel approaches to improve the diagnosis and prognostication of MPM in pleural effusions and peripheral blood samples.

**Methods:**

CellSearch-based and flow cytometry-based assays using melanoma cell adhesion molecule (MCAM) to identify circulating tumor cells (CTCs) in pleural effusions and peripheral blood samples of MPM patients were optimized, validated, explored clinically and, in case of pleural effusions, compared with cytological analyses. Additionally, tumor-associated circulating endothelial cells (CECs) were measured in peripheral blood samples. The assays were performed on a MPM cohort encompassing patients with histology-confirmed MPM (n=27) and in a control cohort of patients with alternative diagnoses (n=22). Exploratory analyses on the prognostic value of all assays were also performed.

**Results:**

The malignancy of MCAM-positive cells in pleural effusions from MPM patients was confirmed. The detection of MPM CTCs in pleural effusions by CellSearch showed a poor specificity. The detection of MPM CTCs in pleural effusions by flow cytometry showed a superior sensitivity (48%) to standard cytological analysis (15%) (*p* = 0.03). In peripheral blood, CTCs were detected in 26% of the MPN patients, whereas in 42% of the MPM patients tumor-associated CECs were detected above the upper limit of normal (ULN). In exploratory analyses the absence of CTCs in pleural effusions, and tumor-associated CECs in peripheral blood samples above the ULN, appeared to be associated with a worse overall survival.

**Conclusion:**

MCAM-based flow cytometric analysis of pleural effusions is more sensitive than routine cytological analysis. Flow cytometric analysis of pleural effusions and tumor-associated CECs in peripheral blood may serve as a promising approach for the prognostication of MPM patients and, therefore, warrants further study.

**Electronic supplementary material:**

The online version of this article (doi:10.1007/s13402-017-0327-7) contains supplementary material, which is available to authorized users.

## Introduction

Malignant pleural mesothelioma (MPM) is an aggressive and treatment-resistant asbestosis-induced neoplasm, of which the incidence is expected to increase in the next years [1]. Diagnosing MPM can be challenging. Especially the distinction between benign and malignant mesothelial proliferation can be extremely difficult [2]. While markers to improve diagnosis such as mesothelin, hyaluronan and osteopontin in plasma and pleural effusions have been described [3–6], they are currently not widely used in the clinic. Furthermore, despite the initially encouraging reports on fibulin-3 [7], the use of this diagnostic marker turned out to be disappointing in clinical practice [8]. MPM patients often present with pleural effusions, but the sensitivity to diagnose MPM using fluid cytology alone varies widely and has been reported to be as low as 26% and as high as 73% [9–11]. While fluid cytology is sometimes used to establish the diagnosis of MPM, performing a pleural biopsy with histological sampling is still recommended by the ESMO Clinical Practice Guidelines to definitively diagnose MPM [12]. A pleural biopsy, either performed by video-assisted thoracic surgery (VATS) or by an open procedure is, however, an invasive procedure with an associated morbidity, and even when adequate tissue is obtained it can be difficult to conclusively diagnose MPM [13].

In addition to the abovementioned difficulties in diagnosing MPM, another clinical challenge is the current lack of robust prognostic or predictive biomarkers for MPM [14], which limits the options to further personalize the treatment of MPM patients. Putative interesting tools to improve the diagnosis and prognostication of patients with MPM include the assessment of (circulating) tumor cells (CTCs) or circulating endothelial cells (CECs) in pleural effusions and/or in peripheral blood.

CTCs are tumor cells that can be detected in the peripheral circulation of patients with solid malignancies, and a robust prognostic value of CTCs has been demonstrated for various tumor types [15–17]. Of the currently available assays for CTC detection, the CellSearch CTC test is the only one that has been approved by the US Food and Drug Administration (FDA). Through this test, tumor cells are isolated by immunomagnetic enrichment from body fluids using ferrofluid nanoparticles coated with epithelial cell adhesion molecule (EpCAM)-specific antibodies. We previously demonstrated that in breast cancer, melanoma cell adhesion molecule (MCAM or CD146) may serve as an alternative marker that is expressed in EpCAM-negative cells [18], and that a modification in the CellSearch CTC enumeration kit can be used to detect MCAM-positive CTCs in breast cancer patients [19]. Expression of MCAM in cytological smears of pleural effusions of MPM patients has been suggested as a novel marker to discriminate between malignant and reactive mesothelium [20]. So far, however, the malignant nature of MCAM-positive cells has not been confirmed, and sensitivity issues are likely to play a role in MPM patients with a low occurrence of tumor cells in pleural effusions when preparing cytological smears. Therefore, the use of a CellSearch MCAM-based enrichment approach may be instrumental to specifically detect MPM cells at low concentrations in pleural effusions and peripheral blood samples of MPM patients.

CECs are endothelial cells that have been sloughed of vessel walls, and have been found to be increased in circulating blood samples of patients with solid malignancies [21, 22]. Recently, we introduced a novel marker, CD276, to distinguish CECs derived from normal endothelium from those that are tumor-derived [23]. Especially since MPM is a well-vascularized tumor and angiogenesis is thought to be important in MPM [24], tumor-associated CECs may serve as a prognostic marker in MPM [25].

This research aimed to optimize techniques to detect tumor cells in pleural effusions and peripheral blood samples of MPM patients, and to obtain more insight into the potential use of CTCs and CECs detected by these techniques as biomarkers in MPM. Data are presented on a cohort of patients with pleural effusions due to MPM and a cohort of patients with pleural effusions due to other causes. In all cases, the pleural effusions were evaluated using MCAM-based methods in conjunction with CellSearch and flow cytometry. Additionally, MPM patient-derived peripheral blood samples were evaluated for the presence of MCAM-positive CTCs and tumor-associated CECs.

## Material and methods

### MCAM expression and recovery of MPM tumor cells

Three primary MPM-derived cell lines (MESO-1, MESO-2, MESO-4; MPM cells isolated from human pleural effusion samples) were stained with MCAM-APC (clone 541–10B2; Miltenyi Biotec GmbH, Bergisch Gladbach, Germany) after which MCAM-positive cells were recovered using a FACS Fortessa flow cytometer (BD Biosciences, San Jose, CA, USA). Subsequently, MCAM expression was quantified relative to unstained cells (signal/noise) using the FCS Express tool (De Novo Software, Los Angeles, CA, USA). To determine the recovery of mesothelioma cells by CellSearch technology (Janssen Diagnostics, Raritan, NJ, USA) using MCAM-based enrichment, 100 cells of the MPM-derived cell lines were spiked into 7.5 ml healthy donor blood in duplo. Subsequently, MCAM-based CTC enumeration was performed as described before [18, 19]. Briefly, to enumerate MCAM-CTCs the anti-EpCAM ferrofluids from the Circulating Epithelial Cell Kit (Janssen Diagnostics) were substituted with anti-MCAM ferrofluids from the Circulating Endothelial Cell Kit (Janssen Diagnostics). Other components of the Circulating Epithelial Cell Kit were left untouched, meaning that cells were stained according to the standard Circulating Epithelial Cell protocol with cytokeratin (CK) 8/18/19, DAPI (for nuclear staining) and CD45 (for exclusion of leukocytes). As an extra marker we used FITC-conjugated CD34 (BD Biosciences, San Jose, CA, USA) to exclude CK18-expressing CECs. MCAM-positive tumor cells were thus defined as CK8/18/19+, DAPI+, CD45- and CD34- after enrichment for MCAM.

### SNP array analysis

Pleural effusions were flow cytometrically sorted using a FACS Aria sorter (BD Biosciences). The following populations were sorted: 1) MCAM+, DRAQ5+ and CD45- cells; 2) MCAM-, DRAQ5+ and CD45- cells and 3) CD45+ cells (leukocyte control; see Supplementary Fig. 1). DNA was isolated from these populations using a Nucleospin DNA kit (Macherey-Nagel, Düren, Germany) and DNA concentrations were quantified using a Qubit dsDNA HS Assay kit (Thermo Fisher, Waltham, MA, USA). Next, DNA from these populations was subjected to SNP array analysis using a CytoScan HD Array Kit (Affymetrix, Santa Clara, CA, USA). The SNP array data were subsequently analyzed for the presence of copy number variations (CNVs) using the Chromosome Analysis Suite (ChAS) software (Affymetrix).

### Patients and inclusion criteria

Two cohorts of patients were included in this prospective study. The first cohort (MPM cohort) consisted of patients with pathology-confirmed MPM or a high suspicion of MPM, presenting with pleural effusions who needed to undergo a pleural drainage or video-assisted thoracoscopy as part of standard care. The second cohort (control cohort) consisted of patients who presented with pleural effusions with a need to drain as part of standard care and in whom an established diagnosis other than MPM was made. In all cases the pleural effusions were sent to a pathological laboratory and processed as a part of standard care. Additionally, in all cases 20 ml of pleural effusion residual material was sent for MCAM-based CTC enumeration and FC. In the MPM cohort also 2 × 10 ml peripheral blood was drawn for MCAM-based CTC enumeration and CEC enumeration. This study ran from March 2014 to January 2016 in two centers in The Netherlands (Erasmus MC Cancer Institute, Rotterdam, The Netherlands and Amphia Hospital, Breda, The Netherlands). All patients provided written informed consent, and the institutional boards of both participating centers approved the protocols (Erasmus MC ID MEC-2014-116; Netherlands Trial Register NTR4575). All procedures involving human participants were in accordance with the ethical standards of the institutional and/or national research committee and with the 1964 Helsinki declaration and its later amendments or comparable ethical standards.

### Processing of pleural effusion and peripheral blood samples

Pleural effusions were processed for standard cytology analysis as part of standard care. All pleural effusion cytology slides were revised by one pathologist (MdB), who is a member of the Dutch national mesothelioma expert pathology panel. Pleural effusions for research purposes were first filtered on a Falcon Cell Strainer (70 uM; Corning Incorporated, Corning, NY, USA) to remove cell clumps and debris and processed within 24 h after the pleural drainage. Peripheral blood was drawn in CellSave tubes, and processed within 96 h for MCAM-CTC enumeration and CEC enumeration.

The MCAM-based CTC enumeration in pleural effusions (3 ml) and peripheral blood samples (7.5 ml) was performed as described above and before [18, 19]. As the CellSearch system requires an area in which red blood cells are present to start processing the sample, a ‘dummy’ tube with the bottom of the tube marked black (marking the area in which packed red blood cells are expected) was used to process the erythrocyte-poor pleural effusions samples on the CellSearch system after centrifugation. CK+, DAPI+, CD45- and CD34- were considered positive events as described before [19]. In case 5 or more cells were closely connected to each other, the event was counted as a cluster.

For flow cytometry of the pleural effusion samples, 10 ml filtered pleural effusion was washed twice in phosphate buffered saline (PBS) and resuspended in 1 ml PBS supplemented with 1% bovine serum albumin. Subsequently, 100 ul of the suspension was stained using our pleural effusion antibody panel encompassing MCAM, pan-cytokeratin (pan-CK), CD45, CD34, two MPM-specific markers thrombomodulin (CD141) and podoplanin (D2–40) and two carcinoma-specific markers CEA (CD66e) and Claudin-4, and DAPI (Supplementary Tables 1 and 2). All antibodies were carefully titrated using positive and negative controls. As DAPI and pan-CK stain intracellular, they were employed after fixation and permeabilization of the cells using a FIX&PERM Cell fixation and permeabilization kit (Nordic-MUbio, Susteren, the Netherlands) according to the manufacturer’s instructions. The samples were loaded on an FACS Fortessa flow cytometer (BD Biosciences) and analyzed in FCS Express (De Novo Software). Cells that were MCAM+, DAPI+, pan-CK+, CD45- and CD34- were considered as putative MPM tumor cells (see gating strategy in Supplementary Fig. 2). When these cells were also negative for the carcinoma-specific markers used, they were considered to be true MPM tumor cells. Positivity was evaluated against unstained controls for each fluorochrome, and positivity for the MPM and carcinoma-specific markers was defined as ≥ 20% of the putative MPM tumor cells being positive for that marker.

The enumeration of CECs was performed in 4 ml peripheral blood as described before [22, 23]. Cells that were CD34+, DRAQ5+, MCAM+ and CD45- were defined as CECs, and CECs also expressing CD276 were defined as tumor-associated CECs.

### Statistical considerations and analyses

Our primary objective was to increase the sensitivity of pleural effusion evaluation in MPM using MCAM-based CellSearch CTC enrichment compared to manual fluid cytology evaluation. Based on reports on the expression of MCAM in both tissue [26] and pleural effusion specimens [20] of MPM patients, which has been reported to be > 80%, we assumed that a sensitivity of at least 80% could be reached using the CellSearch enrichment technology. According to literature data, fluid cytology by a pathologist has a sensitivity of approximately 30%. We powered the study according to a statistical worst-case scenario of discordant proportions of 0.2 and 0.7 using the McNemar test, and 34 patients with a confirmed diagnosis of MPM would be needed to reach significance at a level of α = 0.05 and β = 0.10. Twenty patients with pleural effusion due to another cause than MPM were included to explore the specificity of the test. Secondary objectives of the study were to confirm the malignant nature of MCAM-positive tumor cells in pleural effusions, to develop a flow cytometric strategy on pleural effusions to diagnose MPM, to investigate the presence of CTCs and tumor-associated CECs in peripheral blood samples, and to perform exploratory analyses on the prognostic value of all measured biomarkers in the context on this study. The numbers of cells between the two cohorts were compared using the Mann-Whitney U test, and the presence or absence of a biomarker between the two groups was compared using a χ^2^ test. All reported *p*-values are two-sided, and a significance level α = 0.05 was used. All constructed Kaplan-Meier curves are exploratory as the number of patients and events was low and no formal statistics could be performed on the curves. All data analyses were done using Stata/SE version 12 (StataCorp LP, College Station, TX, USA).

## Results and discussion

MCAM expression was evaluated in three primary MPM-derived cell lines. Two cell lines (MESO-2 and MESO-4) were found to show a high MCAM expression, while one cell line (MESO-3) showed a moderate to low expression. The recovery of mesothelioma cells spiked into peripheral blood samples using the MCAM-based CellSearch technology (performed in duplicate for each cell line) was 48–63% for the two MPM cell lines with a high MCAM expression (MESO-2 and MESO-4) and 4–8% for the MPM cell line with low MCAM expression (MESO-3).

To confirm the malignant nature of MCAM positive cells in pleural effusions, pleural effusions from six patients with a pathology-confirmed diagnosis of MPM were flow cytometrically sorted for subsequent SNP array analysis. In two flow cytometrically sorted MCAM-positive fractions from two separate patients a sufficient amount of DNA was present for reliable SNP array analysis. We found that the MCAM-positive populations (MCAM+, DRAQ5+, CD45-) of both these patients exhibited a number of CNVs, while the leukocyte populations from the same patients did not exhibit these CNVs, as expected (Supplementary Fig. 3). These results demonstrated the putative malignant nature of the MCAM-positive tumor cells in the pleural effusions of the MPM patients and led to the initiation of the clinical study described below.

A total of 49 patients was included. The MPM cohort consisted of 27 patients, and the control cohort of 22 patients (Table 1). The patients in the control cohort represented a mixed population with regard to malignant pleural effusions (52%) and benign pleural effusions (48%). In the MPM cohort, the majority of the patients had an epithelial type MPM (81%). The MPM patients were evenly distributed between stage I-II (52%) and stage III-IV (48%) and most of them (70%) did not receive any prior MPM treatment.

**Table 1:** Baseline characteristics

	MPM cohort (*n* = 27)	Control cohort (*n* = 22)
Diagnosis		
Malignant pleural mesothelioma	27	
Epithelial malignancy		9
Non-epithelial malignancy other than mesothelioma		3
Benign		10
Gender		
Female (%)	1 (4%)	8 (36%)
Age		
Years (range)	70 (27–90)	67 (30–91)
WHO stage		
WHO 0–1	14	11
WHO 2	11	8
WHO 3	2	3
Smoking		
Never	9	6
Past	10	11
Current	8	5
Asbestosis exposure		
No	7	3
Yes	20	6
Unknown	0	13
Pathology		
Epithelial MPM	22	
Biphasic MPM	5	
Stage		
MPM stage I-II	14	
MPM stage III-IV	13	
Prior treatments for MPM		
No	19	
Yes	8	
Currently on treatment for MPM		
No	23	
Yes	4	

Pleural effusions of 41 patients could be evaluated using CellSearch MCAM-based enrichment. Cells meeting our criteria for being tumor cells were detected in 21 of 23 patients (91%) from the MPM cohort and in 17 of 18 patients (94%) from the control cohort. The median number of cells was 416/3 ml in the MPM cohort and 440/3 ml in the control cohort (*p* = 0.86). Cell clusters were present in 15 patients (65%) from the MPM cohort and in 11 patients (61%) from the control cohort (*p* = 0.79).

Using flow cytometry, tumor cells meeting our criteria for being MPM tumor cells (i.e., MCAM+, CK+, DAPI+, CD45-, CD34-, CEA-, Claudin-4-) were detected in the pleural effusions from 12 of 25 patients in the MPM cohort (48%). Also in one patient (6%) from the control cohort these cells were detected, leading to a specificity of 94%. The median number of MPM tumor cells in the MPM patients was 337 (range 23–10,017). No MPM tumor cells were detected in 4 patients with a biphasic MPM histology. In the majority of MPM patients the tumor cells expressed thrombomodulin (92%), whereas podoplanin expression on these cells was observed in the pleural effusion of only one patient (8%). We found that the MPM tumor cells expressed EpCAM in 33% of the cases and GLUT1 in 58% of the cases. Of note, we found that in the control cohort two patients had MCAM-positive cells, not meeting the criteria for MPM tumor cells given a strong expression of the epithelial markers CEA and Claudin-4, suggesting that these might be carcinoma cells. As it turned out, these patients indeed had metastatic epithelial cancers (breast cancer and thyroid cancer, respectively).

**Fig. 1:**
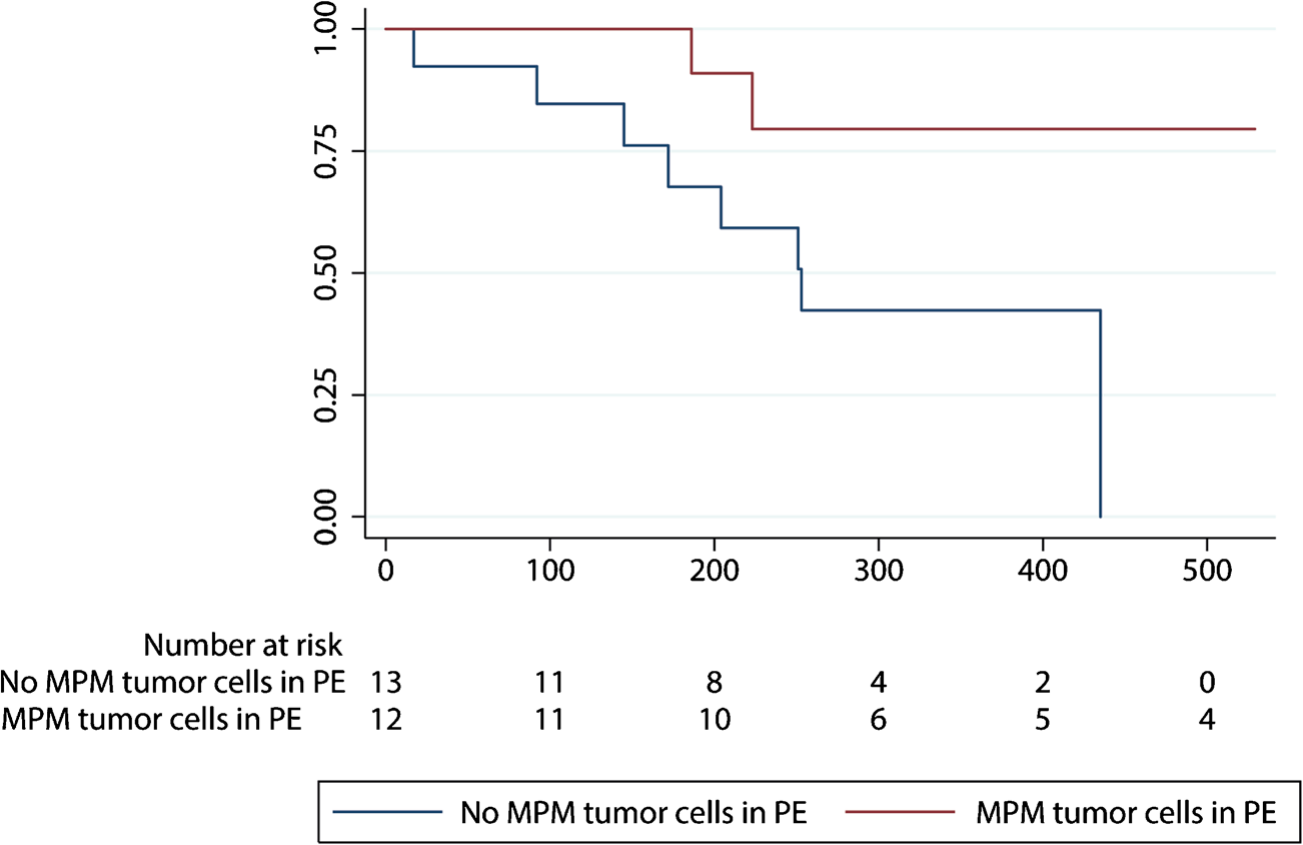
Overall survival according to the detection of tumor cells in pleural effusions using flow cytometry in the MPM cohort

The above flow cytometric observations were compared to those of the pathologist, which is the current clinical standard. Paired observation data were present from 20 patients in the MPM cohort and 18 patients in the control cohort. The pathology review correctly identified 3 MPM patients as having MPM, whereas none of the patients in the control cohort were scored as having MPM, yielding a sensitivity of 15% and a specificity of 100%. The flow cytometric assay identified 6 additional MPM patients compared to those identified by the pathology reviews of the pleural effusions (Table 2, McNemar *p* = 0.03). An exploratory survival analysis revealed that the MPM patients in whom MPM tumor cells were detected using flow cytometry may have a better overall survival (OS) than patients in whom these cells were not detected (Fig. 1).

**Table 2:** Pathology review versus FC assay for patients with MPM in whom matched cytology and FC results from PE obtained on the same day were available

	FC positive for MPM	FC negative for MPM	Total
PA positive for MPM	3	0	3
PA negative for MPM	6	11	17
Total	9	11	20

Circulating tumor cells (CTCs) were detected in 6 of 23 MPM patients (26%). In five patients one CTC per 7.5 ml blood was detected, while in one patient 3 CTCs/7.5 ml were detected. Two patients with CTCs had stage I-II disease, whereas the other 4 patients with CTCs had stage III-IV disease. Four of the patients with CTCs had received prior chemotherapy. Circulating endothelial cell (CEC) enumeration results were available for 24 MPM patients. The median number of CECs was found to be 37/4 ml (range 3–179). The tumor-associated marker CD276 was expressed in a median of 24% (range 7–78%) of the CECs, resulting in 10 patients (42%) having tumor-associated CECs higher than the upper limit of normal (ULN; ≥ 8 tumor-associated CECs/4 ml [23]). Four of these patients had stage I-II disease, and 6 patients had stage III-IV disease.

**Fig. 2:**
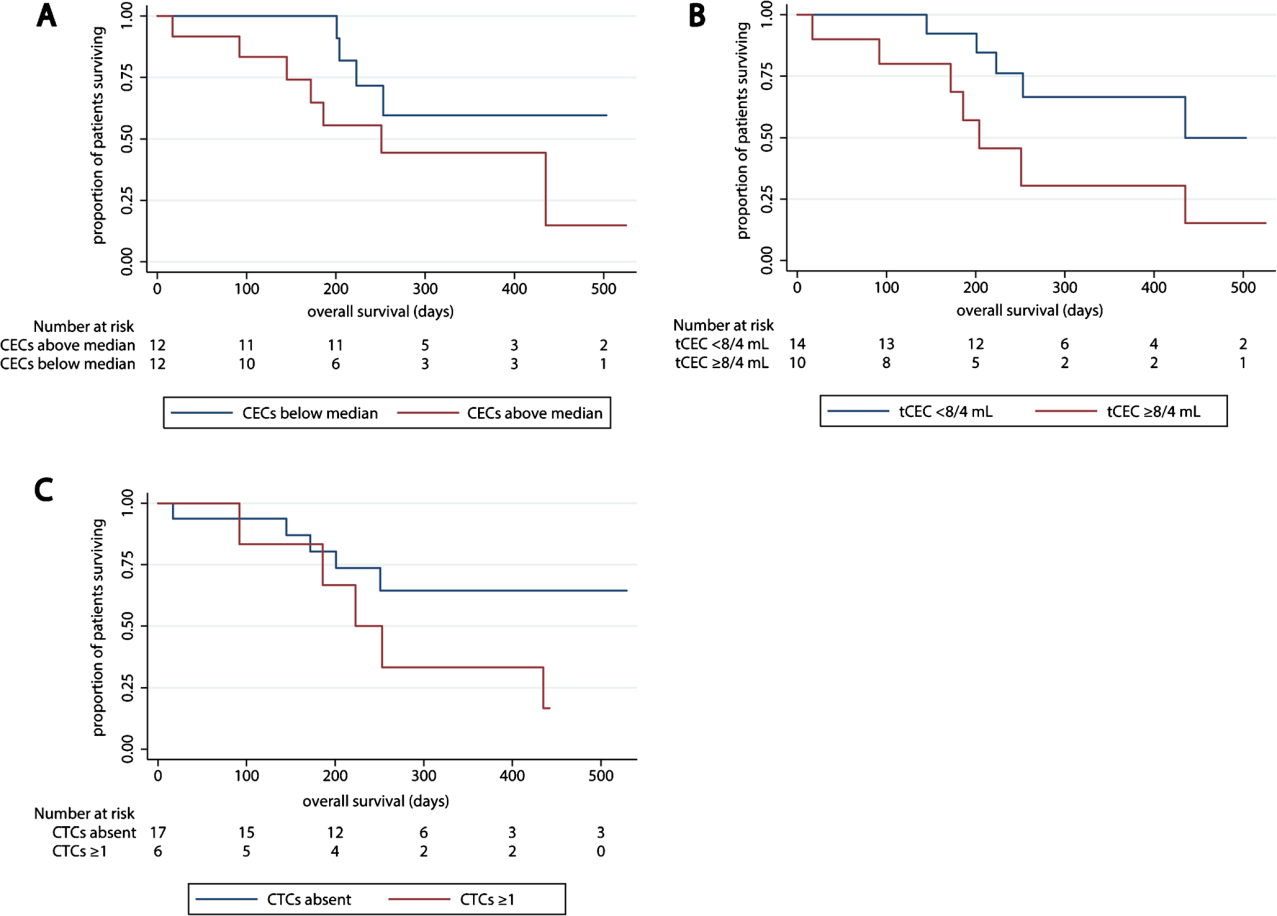
Overall survival in the MPM cohort according to biomarkers in peripheral blood. **a** patients separated by the median number of CECs. **b** patients above and below the upper limit of normal for tumor-associated CECs. **c** overall survival according to the presence of CTCs

Through exploratory survival analyses, we found that the presence of CTCs or a CEC number above the median did not appear to be associated with overall survival (OS). The presence of tumor-associated CECs higher than the ULN may, however, be associated with OS (Fig. 2).

Both the diagnosis and the treatment of MPM patients are hampered by the limited availability of biomarkers that are clinically useful. Here, we presented and validated techniques to identify tumor cells in pleural effusions and peripheral blood samples of MPM patients and, subsequently, explored whether detecting these tumor cells in pleural effusions might improve the diagnosis of MPM, and whether enumerating CTCs and CECs improves the prognostication of MPM patients. We found that enumeration of tumor cells in pleural effusions using the FDA-cleared CellSearch machine provided a limited specificity. Using flow cytometry, a higher sensitivity than with standard pathological assessment was observed, and this sensitivity was accompanied by an acceptable specificity. Additionally, we found through exploratory analyses that flow cytometry-based enumeration of tumor cells in pleural effusions and a similar enumeration of tumor-associated CECs in peripheral blood samples may have prognostic value.

It was hypothesized that the sensitivity and specificity for diagnosing MPM could be improved by enriching for the presumed MPM-specific marker MCAM. The malignant nature of MCAM-positive cells was confirmed using flow cytometric sorting in conjunction with genomic analyses (i.e., SNP-based DNA copy number profiling). The CellSearch system was chosen to enrich and enumerate these cells since MCAM-specific enrichment was already up-and-running on this machine [18, 19]. Using this latter technique, however, we noted that a vast number of non-mesothelioma pleural effusions contained relatively high numbers of cells meeting the criteria for tumor cells. This limited specificity of the CellSearch system is in accordance with observations reported by Lustgarten and colleagues [27]. This group attempted to improve the diagnosis of malignant pleural effusions by the CellSearch system using EpCAM to enrich for CTCs. By doing so, they detected up to 2556 EpCAM-positive cells/3 ml in patients with benign effusions and non-epithelial effusions. Since in the present study high numbers of cells were scored with the CellSearch system in patients who had no detectable MCAM-positive cells using flow cytometry, we hypothesize that the reactive mesothelium may be a-specifically enriched when it is excessively present in the pleural effusions. When one of the pleural effusion samples was treated with immunoglobulins prior to CellSearch enrichment to block a-specific binding of MCAM antibodies to Fc receptors, 60% less cells were observed than in samples not treated with immunoglobulins. These findings suggest that a-specific binding of reactive mesothelium through Fc receptor binding may, at least partly, explain the limited specificity of the CellSearch system. This limited specificity led to an early discontinuation of this part of the study, since obviously the primary study endpoint could not be reached. Using flow cytometry, however, a better specificity than with the CellSearch system was obtained. In addition, we found that the flow cytometry assay exhibited an improved sensitivity over standard cytological analyses. However, where the cytological analyses yielded a 100% specificity, the flow cytometry assay yielded one false-positive finding, leading to a 94% specificity. This latter finding should, however, be interpreted with caution since next to the presence of a benign mesothelial proliferation, this patient was clinically suspect for MPM. During a follow-up period of 1.5 years, however, MPM did not become manifest c.q. was not pathologically confirmed.

In the past years ample research has been devoted to the identification of novel markers to diagnose MPM in pleural effusions, but so far none of the identified markers has been widely incorporated in clinical practice. Recently, others have reported that p16 FISH and BRCA1-associated protein 1 (BAP1) immunohistochemistry-based assays may improve the sensitivity of pleural effusion cytology, with a sensitivity of 45% to 84% for p16 and of 33% to 74% for BAP1 [28]. The sensitivity of the flow cytometric assay used in the current study falls within the range of these assays. In addition, we found that the sensitivity of the flow cytometric assay used is comparable with that of the detection of tumor antigens in pleural effusions as recently evaluated in a meta-analysis [29]. Unfortunately, no cytological slides were available to compare the flow cytometry assay with MCAM (or other marker) staining results. Of note, we found that the numbers of MPM tumor cells in the pleural effusions detected by flow cytometry were often low, indicating that there may be a small chance to detect them through standard cytological analyses.

Surprisingly, we found that patients with MPM tumor cells in their pleural effusions as detected by flow cytometry exhibited an improved OS compared those in which these tumor cells were not detected. There are two possible explanations for this observation: 1) MCAM is a marker for a good prognosis; 2) the presence of MPM tumor cells in pleural effusions is related to an increased chance of response to therapy. Obviously, validation of this preliminary finding and, when confirmed, exploration of the underlying reason in a larger cohort will be necessary.

In a quarter of the patients CTCs were detected in peripheral blood samples using our MCAM-based enrichment method. This CTC-positivity rate was lower than that previously reported by others using standard EpCAM-based CTC enrichment. Yoneda et al. [30] reported that 33% of their MPM patients had EpCAM-positive CTCs, while Raphael et al. [31] detected EpCAM-positive CTCs in 44% of their MPM patients. Since MCAM is more widely expressed than EpCAM in MPM tissues, the lower CTC-positivity rate as observed here is surprising. This discrepancy may be explained by the inclusion of more patients with stage IV disease in both studies evaluating EpCAM-positive CTCs than in the current study. Our observations, along with the observations regarding EpCAM-positive CTCs, suggest that the presence of CTCs is limited in patients with MPM, which is also reflected by the fact that clinically detectable metastasis outside the thorax is a relatively rare and late event in MPM [32]. The presence of MCAM-positive CTCs did not appear to be associated with OS in an exploratory cohort analysis. Also, the presence of CECs above the median did not appear to be related to OS. Only when tumor-associated CECs were analyzed they seemed to be of prognostic value for OS. Yoneda et al. [25] previously reported a modest relation between poor OS and high CEC numbers in a cohort of 79 MPM patients. Our findings on a much smaller cohort of patients suggest that when a marker specific for tumor-associated CECs is added, i.e., excluding CECs derived from the normal vasculature [33], the prognostic power of CECs may improve.

In conclusion, we show in this exploratory study that MCAM-positive cells in pleural effusions of patients with MPM are malignant in nature, and that several biomarkers may be used to improve the diagnosis and prognostication of patients with MPM. The enumeration of tumor cells and tumor-associated CECs may lead to an improvement of both. Our results warrant a further investigation of larger MPM cohorts.

## Electronic supplementary material

ESM 1 (DOCX 1091 kb)
