# Supplementary material for: Improved diagnosis and prognostication of patients with pleural malignant mesothelioma using biomarkers in pleural effusions and peripheral blood samples – a short report
Source: Cell Oncol (Dordr). 2017 Jun 2;40(5):511–9. doi: 10.1007/s13402-017-0327-7 (PMC5608799; doi:10.1007/s13402-017-0327-7)
Supplement: Supplementary file 1 — (DOCX 1091 kb) [file 13402_2017_327_MOESM1_ESM.docx]

**Supplementary Figure 1. Gating strategy for flow cytometric sorting.** Nucleated cells (DRAQ5-positive) were sorted according to their CD146/MCAM and CD45 expression. Gate 3 represents MCAM+ DNA+ putative tumor cells. Gate 4 represents MCAM- DNA+ cells, who appeared to have low side scatter and forward scatter, which might indicate that these cells are near-apoptotic. Gate 2 represents CD45+ MCAM- cells, which is the leukocyte control. Of note is that a subset of leukocytes express MCAM, which are activated leukocytes (Elshal et al., Blood 2005).

**
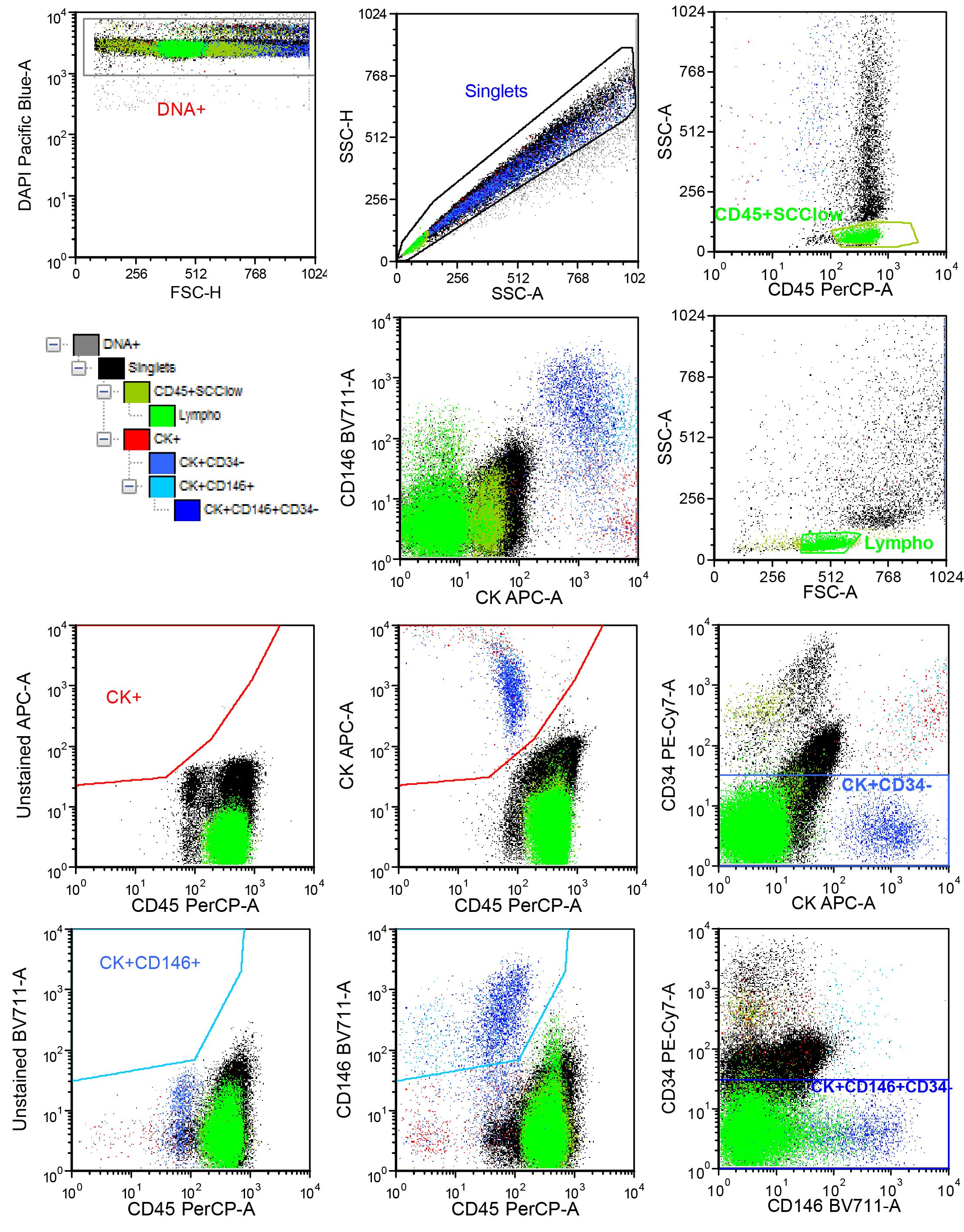

Supplementary Figure 2. Gating strategy (continued on next page)**

**
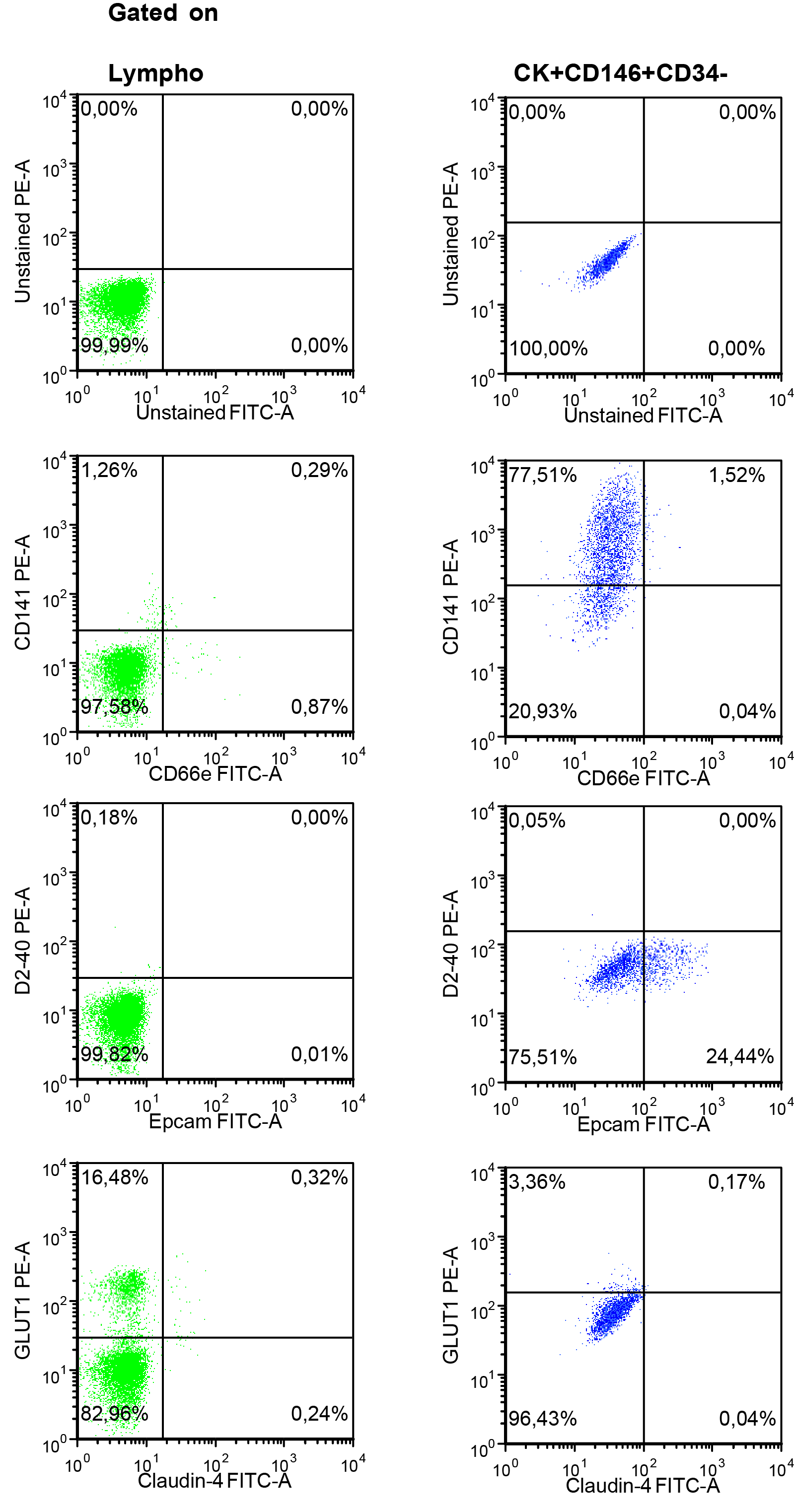
**

**Supplementary Figure 2. Gating strategy.** All DNA+ cells (DAPI-positive) and singlets were selected for further analysis. Lymphocyte control population was defined as CD45+ and side scatter low. Cytokeratin (CK) and MCAM (CD146) expression were measured against an unstained control. All CK+ and MCAM+ cells were then further characterized for CD34, too exclude possible CD34-positive endothelial cells. All DNA+ CK+ MCAM+ CD34- cells (depicted in blue on the second page), dubbed putative MPM tumor cells, were subsequently characterized for 6 additional markers. Positivity for a marker was defined as ≥20% of the putative MPM tumor cells being positive for that marker. In this example, the putative MPM tumor cells are positive for CD141 (thrombomodulin) and EpCAM, and negative for carcinoma markers (Claudin-4 and CD66e/CEA).

**
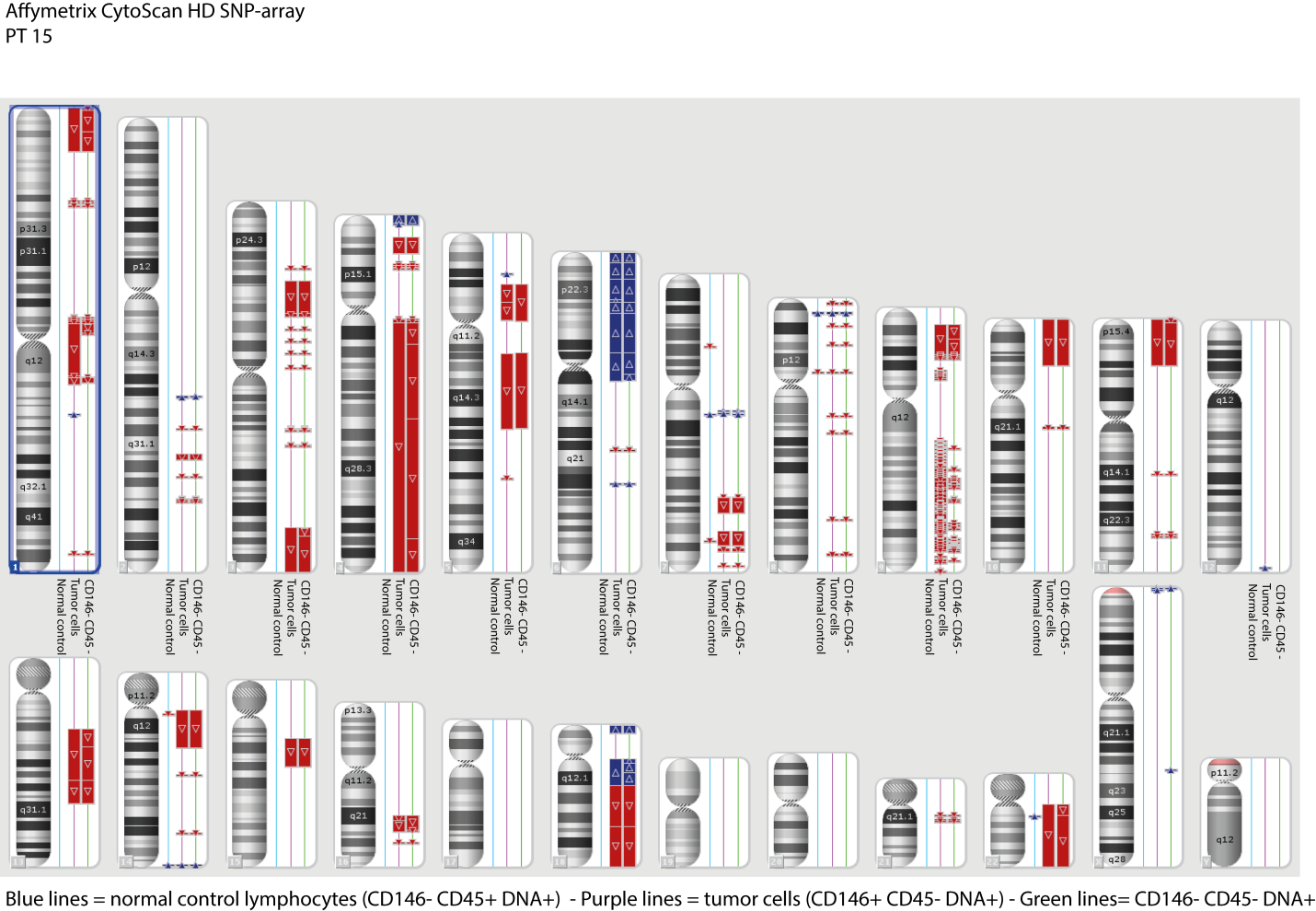
**

**Supplementary Figure 3. SNP arrays of gated populations presented in Supplementary Figure 1.** Blue lines represent the normal control lymphocytes (CD146-neg, CD45-pos, DNA+; gate 2), Purple lines represent putative tumor cells (CD146-pos, CD45-neg, DNA+; gate 3), and green lines represent a CD146-neg, CD45-neg, DNA+ population (gate 4). Red bars indicate losses, and blue bars indicate gains.

| **Supplementary Table 1. Flow cytometric panel for pleural effusion** | | | | | | | |
| --- | --- | --- | --- | --- | --- | --- | --- |
| Tube | **Pacific blue** | **Brilliant Violet 711** | **FITC** | **PE** | **PerCP** | **PE-Cy7** | **APC** |
| 1 | DAPI* | CD146 | unstained | unstained | CD45 | CD34 | unstained |
| 2 | DAPI* | unstained | unstained | unstained | CD45 | CD34 | pan CK* |
| 3 | DAPI* | CD146 | unstained | unstained | CD45 | CD34 | pan CK* |
| 4 | DAPI* | CD146 | CD66e | CD141 | CD45 | CD34 | pan CK* |
| 5 | DAPI* | CD146 | EpCam | D2-40 | CD45 | CD34 | pan CK* |
| 6 | DAPI* | CD146 | Claudin-4 | GLUT1 | CD45 | CD34 | pan CK* |

*: intracellular staining after fixation and permeabilization

| **Supplementary Table 2. Details of flow cytometric panel for pleural effusions** | | | | | | |
| --- | --- | --- | --- | --- | --- | --- |
| **Antibody** | **Alternative name** | **Fluorochrome** | **Company** | **Clone** | **Used dilution** | **Comments** |
| DAPI |  | Pacific Blue | Janssen Diagnostics |  | 1:10 | from CellSearch CTC kit |
| MCAM | CD146 | Brilliant Violet 711 | BD Biosciences | P1H12 | 1:10 |  |
| CEA | CD66e | FITC | BioConnect | MCA1744F | 1:1 |  |
| EpCAM | CD326/Ber-EP4 | FITC | BD Biosciences | EBA-1 | 1:1 |  |
| Claudin-4 |  | FITC | R&D systems | 38231 | 1:2 |  |
| Thrombomodulin | CD141 | PE | Miltenyi Biotec | AD5-14H12 | 1:10 |  |
| Podoplanin | D2-40 | PE | eBioscience | 8.1.1 | 1:20 |  |
| GLUT1 |  | PE | R&D systems | 202915 | 1:1 |  |
| CD45 |  | PerCP-Cy5 | BD Biosciences | 2D1 | 1:1 |  |
| CD34 |  | PE-Cy7 | Beckman Coulter | 581 | 1:2 |  |
| Pan-cytokeratin |  | APC (eFluor 660) | eBioscience | AE1/AE3 | 1:10 |  |
